# Suitability of DNN-based vessel segmentation for SIRT planning

**DOI:** 10.1007/s11548-023-03005-x

**Published:** 2023-08-03

**Authors:** Farina Kock, Felix Thielke, Nasreddin Abolmaali, Hans Meine, Andrea Schenk

**Affiliations:** 1 Fraunhofer Institute for Digital Medicine MEVIS, Max-von-Laue-Str. 2, Bremen, 28359 Germany; 2 Diagnostic and Interventional Radiology and Nuclear Medicine, St. Josef-Hospital, University Hospitals of the Ruhr University of Bochum, Gudrunstr. 56, Bochum, 44791 Germany

**Keywords:** Liver vessels, Hepatic arteries, Segmentation, Deep learning, CT

## Abstract

**Purpose:**

The segmentation of the hepatic arteries (HA) is essential for state-of-the-art pre-interventional planning of selective internal radiation therapy (SIRT), a treatment option for malignant tumors in the liver. In SIRT a catheter is placed through the aorta into the tumor-feeding hepatic arteries, injecting small beads filled with radiation emitting material for local radioembolization. In this study, we evaluate the suitability of a deep neural network (DNN) based vessel segmentation for SIRT planning.

**Methods:**

We applied our DNN-based HA segmentation on 36 contrast-enhanced computed tomography (CT) scans from the arterial contrast agent phase and rated its segmentation quality as well as the overall image quality. Additionally, we applied a traditional machine learning algorithm for HA segmentation as comparison to our deep learning (DL) approach. Moreover, we assessed by expert ratings whether the produced HA segmentations can be used for SIRT planning.

**Results:**

The DL approach outperformed the traditional machine learning algorithm. The DL segmentation can be used for SIRT planning in $61\%$ of the cases, while the reference segmentations, which were manually created by experienced radiographers, are sufficient in $75\%$. Seven DL cases cannot be used for SIRT planning while the corresponding reference segmentations are sufficient. However, there are two DL segmentations usable for SIRT, where the reference segmentations for the same cases were rated as insufficient.

**Conclusions:**

HA segmentation is a difficult and time-consuming task. DL-based methods have the potential to support and accelerate the pre-interventional planning of SIRT therapy.

## Introduction

With increasing numbers, liver cancer is currently among the top five causes of cancer-induced deaths in 90 countries worldwide [1]. The geometry of the vascular systems in the liver is important for diagnosing, pre-interventional planning and treatment of liver diseases. For example, segmentation of the hepatic arteries (HA) is of high interest in selective internal radiation therapy (SIRT), a treatment modality for malignant tumors in the liver that aims at irradiating the tumor locally by inserting small radioactive beads. Since liver tumors are mostly supplied by arterial blood, these beads are inserted by placing a catheter through the aorta into the tumor-feeding arteries. To plan this procedure, a contrast-enhanced computed tomography (CT) is acquired so that the arteries can be visualized and segmented. In addition, the portal (PV) and hepatic veins (HV) can be segmented in a delayed contrast phase.

**Table 1:** Related works on liver vessel segmentation sorted by relevance

Method	Year	Vessels	Modality	Segmentation Method
Wang et al. [8]	2016	HA	CTA	Machine learning
Cheng et al. [6]	2015	HA	CTA	Active contour model
Friman et al. [7]	2010	HA	CTA	Tracking
Badoual et al. [11]	2016	HA	MRA	Tracking
Kazami et al. [12]	2022	PV, HV	CT	Deep learning
Svobodova et al. [13]	2022	PV, HV	CT	Deep learning
Yan et al. [14]	2021	PV, HV	CT	Deep learning
Keshwani et al. [15]	2020	PV, HV	CT	Deep learning
Xu et al. [16]	2020	PV, HV	CT	Deep learning
Kitrungrotsakul et al. [17]	2019	PV, HV	CT	Deep learning
Yu et al. [18]	2019	PV, HV	CT	Deep learning
Ibramigov et al. [19]	2018	PV	CT	Deep learning
Huang et al. [20]	2018	PV, HV	CT	Deep learning

It is important to note that timing of the contrast agent administration is crucial and the appearance of the different hepatic vessel systems is variable. Usually, it takes 15–35 s for the contrast agent to reach the HA and 60–70 s until it reaches the hepatic veins after injection [2]. When CT scans are acquired in the arterial phase, the parallel PV often shows enhancement as well, making it difficult to distinguish the vasculature based on voxel values alone.

In the past, different methods have been introduced to segment vessel structures in medical images such as image filtering and enhancement, tracking approaches, deformable models, and machine learning. Profound research on vessel segmentation has been undertaken in the field of the retina and the heart [3]. In the liver, vein segmentation (HV and PV) has also been studied in detail [4], but automatic extraction of the HA system is rare. A possible reason is the increased difficulty of this task, since the arteries are comparatively small in diameter (0.5 cm) and have variations in their anatomical structure in about one third of the individuals [5].

Existing HA segmentation methods and DL-based PV and HV algorithms are listed in Table  1. While traditional methods often rely on initial constraints, such as seed points, DL models are data-driven and require large amounts of data. HA segmentation developed either with DL or with traditional image processing techniques is an uncommon research topic. Cheng et al. [6] developed a constrained B-snake algorithm and tested it on three CT angiography (CTA) images for HA segmentation. On average, they achieved a Jaccard coefficient of 0.86 compared to ground truth segmentation of two experienced radiologists. Friman et al. [7] also used CTA images to test their semi-automatic segmentation to extract the HA. Their algorithm requires a manual seed point to be set, which is used to initiate a region growing algorithm followed by a multiple hypothesis template tracking.

Furthermore, Wang et al. [8] introduced a segmentation approach for multi-phase liver CT using a trained classifier based on vesselness filters, directional dilation and connectivity analysis, which also requires a manually set seed point. They evaluated their algorithm on 18 CTA images obtaining on average a skeleton coverage of 0.55 and a mean symmetric distance of 12.7 mm. Since the algorithm was developed for abdominal CT data of the arterial contrast agent phase and the original source code was available in-house, we decided to use Wang et al.’s method for comparison.

Within the field of DL, we build upon prior work, in which we used 3D convolutional neural networks (CNNs) to segment arteries in abdominal CT images [9] and post-process the output such that a fully connected vessel tree is obtained [10]. To the best of our knowledge, no other research on DL-based HA segmentation has been published yet.

Since manual segmentations of vessels are time consuming and challenging—and thus not fit for clinical routine—, a clinical evaluation of whether automated segmentations are suitable for pre-interventional planning is of high interest. In this work, we evaluate whether the HA vessel trees extracted from CTs using automatic algorithms can be used for pre-interventional planning of SIRT according to a clinical expert. In our evaluation, we compare the results of a traditional image processing algorithm, a DL approach and a manual expert segmentation.

**Fig. 1:**
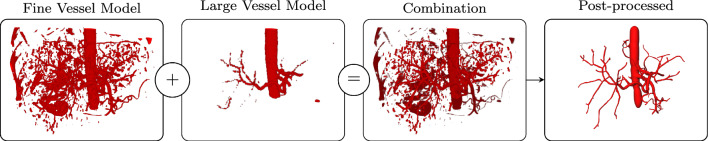
Workflow for DL-based vessel segmentation

## Materials and methods

In the following, we describe the two algorithms we used for segmenting the HA, the dataset, the evaluation metrics, and the clinical evaluation.

### Deep learning based vessel segmentation

The vessel segmentation was developed with the use of aU-Nets that were trained on manually created reference segmentations. The aU-Net is a modified 5-level 3D U-Net [21] in which all 3D convolutions were split into 2D in-plane and 1D across-plane convolutions. The first two levels only contain 2D convolutions and pooling layers, so that the receptive field is of anisotropic size [22]. We developed two models: one is a sensitive model that incorporates a weighted Dice loss and is trained on a fine voxel resolution of (0.8 mm$$)^3$$. The other is a model specifically designed for accurately segmenting large vessels, trained on a coarser resolution of (2.0 mm$$)^3$$. The final output is a combination of these two models followed by a post-processing algorithm [10] to generate a connected vessel tree. The post-processing algorithm is applied onto the output of the joined models and uses the Dijkstra algorithm to find the shortest path for each component to the root point, where the inverse raw model output is used as weights. As root point, the skeleton with the largest associated radius in the output from the large vessel model is used, which usually is a point in the aorta. The workflow is illustrated in Fig. 1.

### Vesselness based vessel segmentation

We applied a HA segmentation algorithm by Wang et al. [8] to compare our DL-based approach with a traditional machine learning method. Wang et al. first apply a Hessian-based vesselness filter to arterial phase CTs. The resulting vesselness response is then processed by a Bayesian classifier to identify the most likely vessel structures. Afterwards a directional dilation operator is used so that missing vessel fragments could be filled. Eventually, disconnected fragments are connected by using a connected component analysis and a vessel connectivity analysis to reconstruct a fully connected 3D vessel tree. Note that this algorithm requires user interaction by setting an initial seed point. To make it fully automatic, we added a heuristic to select a seed point in the same way as for our DL-based method. However, in case this heuristic fails, we set a manual seed point.

### Data

In order to train the DL algorithm, two internal datasets are used. The Yokohama dataset (Yokohama City University, Yokohama, Japan) consist of 112 abdominal CT scans from the arterial phase and the SIRTOP dataset (Dresden University Hospital, Dresden, Germany) contains 100 CT scans from the arterial phase. For both datasets, reference segmentations were produced by highly experienced radiographers. The reference segmentations from Dresden were approved by a senior physician.

Our aim is to clinically evaluate the results on SIRTOP data. We use the Yokohama dataset for pretraining a DNN model. Then, we split the SIRTOP dataset into training, validation and three independent test sets with 12 cases each. Using the pre-trained weights from the Yokohama dataset, we train three models in such a way that each model is assigned one of the test sets while the remaining two test sets were added to the training data. This cross-validation allows us to have sufficient training data along with enough test cases for clinical evaluation.

### Evaluation metrics

The results obtained from the respective vessel segmentation algorithm are evaluated against the corresponding reference segmentations. Since we are more interested in the correctness of the course of the vessels than in their exact diameters, we use the centerline $\text {F}_1$ score (clF1-Score) [23] and its components topology sensitivity (clRecall) and topology precision (clPrecision). To obtain meaningful measurements for the thin arteries, the prediction is dilated by 2.0 mm for the calculation of clRecall, and the reference is dilated by 2.0 mm for the clPrecision. Moreover, since Wang et al.’s method does not produce a fully segmented aorta, we calculate the metrics only within the liver area to obtain comparable results.

**Fig. 2:**
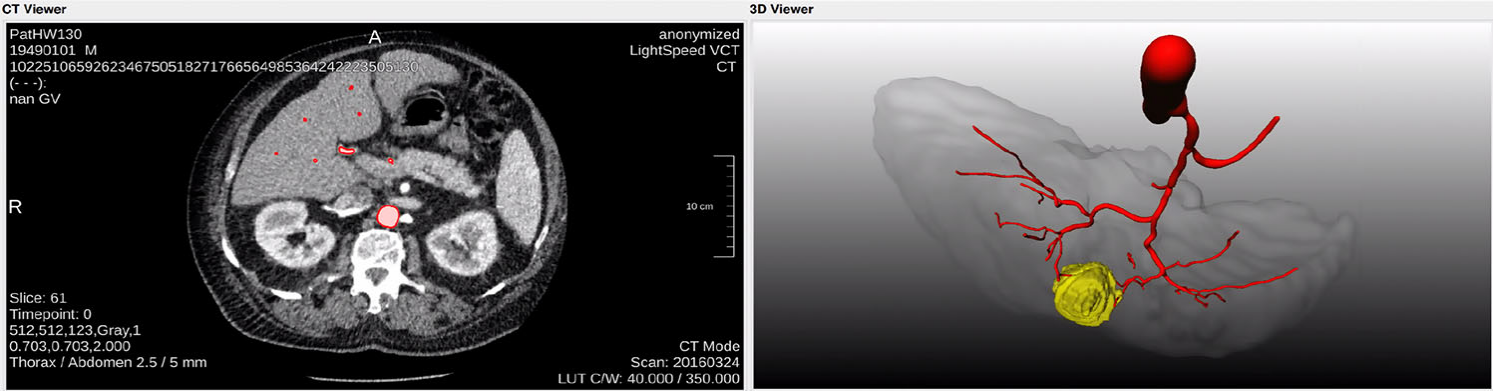
Visual display of vessel segmentation for rating

### Clinical evaluation setup

To assess the quality of the segmentations, we developed a simple rating tool as MeVisLab^1^ application. For each case, the following criteria are assessed:

- image quality (3-point scale: $-\,\circ \,+$)
- segmentation quality (5-point scale: $-\hspace{-2.5pt}- -\, \circ \, +\, +\hspace{-2.5pt}+$)
- usability for SIRT (boolean: yes or no)

As basis for decision-making, the user is offered an interactive axial 2D view of the CT scan as well as the respective 3D model of the liver and the vessel system as shown in Fig. 2. Moreover, the user can enable or disable whether the vessel system, liver contour and—if available—tumor contour shall be displayed. To obtain a fair comparison between the two automatic approaches and the manual segmentations, we randomly shuffled all cases and segmentation methods in the evaluation setup presented to the user.

## Results

Firstly, automated vessel segmentations were evaluated against the reference segmentations that were created by experienced radiographers by calculating centerline-based metrics as described in “Evaluation metrics” section. Secondly, all vessel segmentations (including reference) were rated by a senior physician with over 20 years of experience. We assessed segmentation quality, image quality and usability for SIRT planning.

### Evaluation of centerline metrics

**Table 2:**
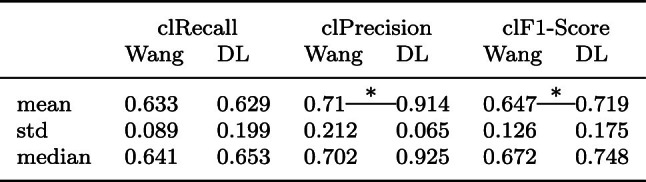
Centerline metrics calculated only within the liver for Wang et al. compared to our DL method

To guarantee comparability, only 29 test cases for which both methods found vessels inside the liver were used

*Statistically significant (Wilcoxon signed rank test with $p < 0.05$)

**Fig. 3:**
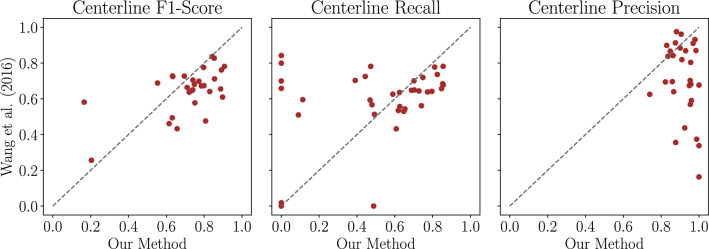
Comparisons of Wang et al.’s method with our DL method on 36 cases

As can be seen in Table 2, Wang et al.’s algorithm and our DL method achieved similar results for clRecall, i. e. both algorithms were able to detect a similar proportion of the arteries present in the reference segmentation. However, while Wang et al.’s algorithm failed to find vessels inside the liver for two test cases (for one case it was not possible to create a segmentation mask at all), our HA segmentation pipeline failed to find vessels inside the liver mask for six cases. clPrecision shows higher scores for the DL approach with a difference of 20.4 percentage points. In total, this leads to a higher clF1-Score for the DL approach with 0.719 versus 0.647. Statistical significance is shown for clPrecision and clF1-Score with a Wilcoxon signed-rank test (based on 29 cases and *p*$<0.05$). Figure 3 also illustrates the per case evaluation metrics. Regarding the per case clRecall, it can clearly be seen that eight cases are zero or close to zero for the DL method and thus do not represent the distribution of the remaining cases.

To conclude from the evaluation of the metrics, Wang et al.’s method is more robust than our DL pipeline failing for only 2 cases compared to 6. Both methods find similar amounts of vessel structures that are part of the reference segmentation. However, Wang et al.’s method produces more false positives and thus has less precision than the DL method.

### Evaluation of clinical rating

We assessed image quality, segmentation quality and usability for SIRT for the different vessel segmentations. It is important to note that Wang et al.’s algorithm failed for one case completely independent of where we manually placed a seed point. Thus, the total number of cases per method differs between 35 (Wang et al.) or 36 (reference and our DL method, respectively).

#### Image quality

**Fig. 4:**
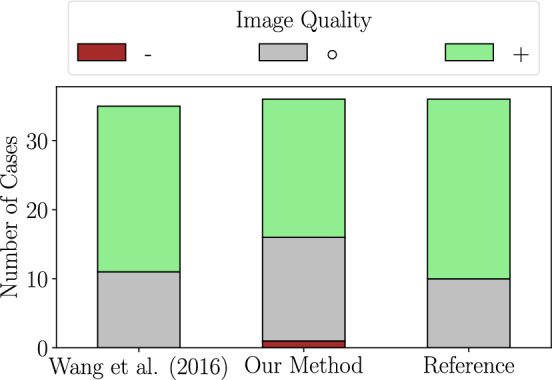
Image quality

The image quality was rated as 3-point-scale ($-\,\circ \,+$). Given the fact that each CT image was rated three times displaying a different vessel segmentation (reference, our method, Wang et al.), the intra-reader variability can be observed. In total, only 19 out of 35 images’ qualities were always rated the same. Moreover, Fig. 4 illustrates the image quality rating as comparison between the three presentations. It can be seen that fewer cases received a $+$ as image quality when the DL segmentation was shown.

**Fig. 5:**
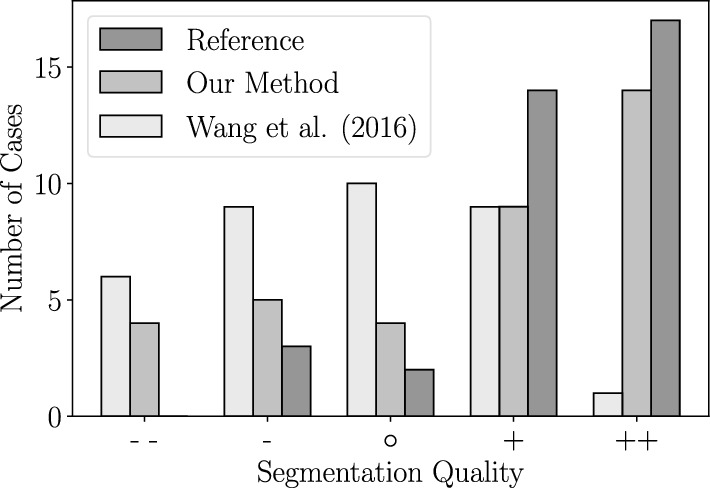
Segmentation quality

#### Segmentation quality

The segmentation quality was rated as 5-point-scale ($-\hspace{-2.5pt}- -\, \circ \, +\, +\hspace{-2.5pt}+$). Wang et al.’s approach achieved a median segmentation quality of $\circ$, while the reference and the DL method achieved a $+$. Wang et al.’s approach achieved segmentation quality results higher or equal than the reference segmentation in $14.29\%$ of the cases (5 out of 35 cases). In contrast, our DL approach performed higher or equal than the reference in $58.33\%$ of the cases (21 out of 36 cases). The distribution of segmentation quality results can be seen in Fig. 5.

#### Usability for SIRT

Whether a segmentation is usable for SIRT planning was rated by “yes” or “no” in the rating tool. While $25.71\%$ (9 out of 35) cases from Wang et al.’s approach were usable for SIRT, a total $61.11\%$ (22 out of 36 cases) of the DL segmentations were usable. From the manually annotated reference segmentations $75\%$ were usable (27 out of 36 cases) according to the expert. It is interesting to note that in two cases the results of our DL method were found to be useable, while the reference segmentations were not.

## Discussion

The localization of hepatic arteries is a time-consuming and crucial task for pre-interventional planning of SIRT. The segmentation of the vessels needs to be of high quality and depth since it is important to see which branches of the artery supply the tumors with blood. Interestingly, only $75\%$ of the manually created reference segmentations were in fact usable for SIRT planning. This shows that manual reference segmentations, which are often considered as *ground truths*, cannot be regarded as perfect annotations.

**Fig. 6:**
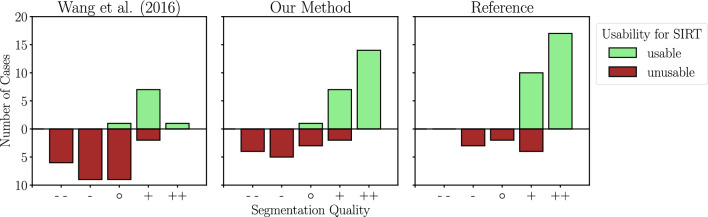
Segmentation quality and its usability for SIRT planning

**Fig. 7:**
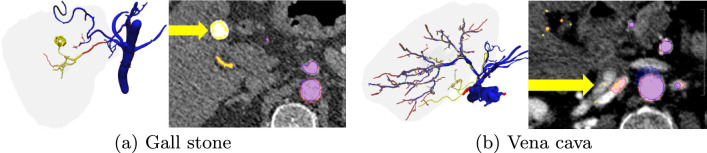
Other structures found by Wang et al.’s method. Red denotes reference segmentation, blue DL method and yellow Wang et al

The observation that our reference segmentations are not always *perfect* has two major implications: First, this clearly affects the evaluation and interpretation of quantitative metrics such as clPrecision or clRecall. Visual inspection is indispensable in order to decide whether a false positive is, for example, truly a false positive or has simply been forgotten in the reference annotation. This becomes increasingly important in challenging tasks such as HA segmentation, in which arteries might consist of only few pixels and are difficult to recognize. Secondly, we also have to be aware of the fact that we train our deep neural networks with these *imperfect* reference segmentations and penalize false predictions (false positives and negatives) in loss functions such as Dice coefficient during training. Thus, deep neural networks might learn to imitate human annotators instead of solving the task of vessel segmentation. This is the reason why we decided to combine a certain *large* vessel model with a model that is considered to be *fine* offering many possible fragments as HA (see Fig. 1).

Since it is not sufficient to assess the quality of an automatic segmentation and its usability for a particular task solely based on evaluation metrics such as clRecall or clPrecision, the relationship between the segmentation quality rating and the centerline metrics is further investigated. Even though Wang et al.’s algorithm receives similar results for clRecall compared to our DL method (see Table 2), their metrics are not represented by their segmentation quality rating. Figure 6 shows that Wang et al.’s method produces many vessel segmentations with negative segmentation quality ratings, which are also not sufficient for SIRT pre-interventional planning. While Pearson’s correlation coefficient (*r*) shows strong correlation for clRecall and the segmentation quality ratings for our DL approach ($r=0.95$), weak correlation can be observed for Wang et al.’s method. Thus, the personal opinion of the physician, as measured by the segmentation quality rating, cannot be fully reflected by the centerline metrics for Wang et al.’s algorithm.

Analyzing the relationship between segmentation and image quality does not show any correlation (Pearson’s correlation coefficient $r=-0.04$). This implicates that we can further interpret segmentation quality independent of the rated image quality. However, to achieve satisfactory segmentation results, the CT scan must meet certain requirements. Due to the small diameter of the HA, high resolution is essential and slice thickness should not exceed about 2 mm. Since Wang et al.’s approach uses a fixed voxel resolution of (0.5  mm )^3^, the results look distorted along the z-axis if slice thickness is too high.

**Fig. 8:**
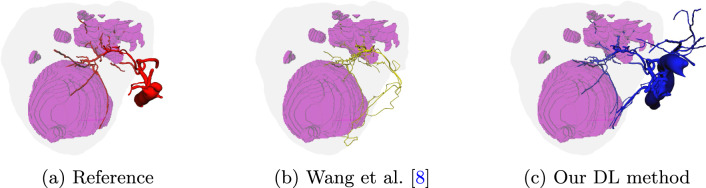
Example in which one HA branch not included in the reference segmentation that supplies a tumor (pink) was found by both methods (**b** and **c**)

A visual inspection of the vascular trees demonstrates a disadvantage of Wang et al.’s traditional image processing technique. Since this method is strongly sensitive to image intensities, it may not learn to differentiate between HA and other enhancing structures such as the vena cava, PV or gall stones as shown in Fig. 7. The DL method, however, has the possibility to learn to distinguish vessels from liver parenchyma. Nonetheless, the *fine* vessel DL model is also more sensitive to all kinds of enhancing structures and might introduce errors as well. We noticed, for example, that in one case the kidney was also segmented and the post-processing algorithm did not succeed in filtering it out since it was connected with the aorta. Figure 8 illustrates one test case, where both algorithms succeeded in recognizing norm variants, where the tumor artery arises directly from the celiac trunk. Moreover, visual inspection shows that Wang et al.’s method does not produce a complete volumetric segmentation for the aorta (see Fig. 8). This type of visualization makes it difficult for a human to follow the course of vessels from thick to thin, and therefore may have a negative impact on the segmentation quality score of the algorithm.

Eventually, investigating the six HA segmentations that were produced with our DL algorithm that did not contain any vessel segmentation within the liver ($\text {clRecall} = 0$), we noticed that our raw DL model output contained many arterial branches within the liver, which the post-processing algorithm filtered out. The reason for that was that the post-processing algorithm failed to overcome the gap between the HA trunk and its split into the left and right HA. By increasing the maximum connection costs in our cost-based post-processing algorithm, the output could strongly be improved.

## Conclusion

In this work, we evaluate the suitability of a DNN-based vessel segmentation for SIRT planning. We show that automatic HA segmentation can support SIRT planning: our DL segmentation was rated usable for SIRT in $61\%$ of the cases. Furthermore, it is important to acknowledge that the manual reference segmentation was also only rated usable in $75\%$ of the cases.

The post-processing algorithm in our method introduced several new failure cases. Future improvements of this step should allow to considerably increase the robustness and applicability of our method. Further research should aim to understand the reasons for the imperfect reference segmentations, which may be due to insufficient image quality. Moreover, given that the reference annotations are imperfect, we want to analyze the false positives of the algorithm output and verify whether they are actually true positives that can be added to our training data. Improved labels may enhance our DL algorithm and further increase the applicability for SIRT planning. Taking into account all the aforementioned optimizations, the ultimate goal would be to conduct a comprehensive study with the aim of evaluating the clinical usability of HA segmentation algorithms on a larger scale.

## Footnotes

1 MeVisLab (https://www.mevislab.de/) is an environment for modular software development with a focus on medical image processing research and development by MeVis Medical Solutions.
